# Clinical Audit of Acute Oxygen Therapy: Enhancing Patient Care at Atbara Teaching Hospital

**DOI:** 10.7759/cureus.91472

**Published:** 2025-09-02

**Authors:** Abubakr Muhammed, Mustafa Awad, Alaa Abbas, Essra Hassan Adam Alradi, Rawan Salah Mohamed Taha, Wishah Busati Saad Mohamed, Reham Abdelgadir Abdelhafeez Mohammed, Hashim Faiez Khader Baket, Tayseer Salih Mohamed Salih, Mohammed Osman Ahmed Osman, Doaa Mahmoud Akasha Mohamed, Ahmed Nabil Mohamed, Abeer Hussien Musa Mohamed, Samir Khedir Bahageil Ibrahim, Mawada M Ali Mohamed, Saad Adam Abdallah Abakar, Mohamed Abdalla Elawad Wedatalla, Musab Alhadi Bushra Alneama, Mohammad Alzain Adam

**Affiliations:** 1 Radiology, University of Gezira, Wad Madani, SDN; 2 Internal Medicine, Sudan Medical Specialization Board, Khartoum, SDN; 3 Anesthesiology, University of Gezira, Wad Madani, SDN; 4 Emergency Medicine, Atbara Teaching Hospital, Atbara, SDN; 5 Anesthesia, ICU, and Perioperative Medicine, Hamad Medical Corporation, Doha, QAT; 6 Internal Medicine, The National Ribat University, Khartoum, SDN; 7 Pediatrics, University of Gezira, Wad Madani, SDN; 8 Critical Care Medicine, Salma Rehabilitation Hospital, Abu Dhabi, ARE; 9 General Surgery, Ribat University Hospital, Khartoum, SDN; 10 Internal Medicine, Mullingar Hospital, Mullingar, IRL; 11 Medicine, Alneelain University, Khartoum, SDN

**Keywords:** clinical audit cycles, documentation standards, oxygen therapy compliance, staff education intervention, weaning and discontinuation protocols

## Abstract

Background: Acute oxygen therapy is vital for managing critically ill and hypoxemic patients, with British Thoracic Society (BTS) guidelines emphasizing precise prescribing, target saturation ranges, and continuous monitoring. Despite clear protocols, global audits reveal frequent lapses in documentation, prescription clarity, and adherence to saturation targets. The objective of this audit was to evaluate and improve the practice of oxygen therapy delivery in the emergency department at Atbara Teaching Hospital, Atbara, Sudan, through baseline assessment, implementation of a standardized protocol, and targeted educational sessions, with reassessment in a follow-up cycle to measure impact.

Methods: A mixed retrospective-prospective audit was conducted over two cycles (Cycle 1: 15^th^ September to 15^th^ October, 2024; Cycle 2: 10^th^ June to 24^th^ June, 2025). Thirty patient records per cycle were assessed against BTS standards, including correct oxygen delivery modes/flow rates, documentation of saturation, target specification (88% to 92% for type 2 respiratory failure), weaning protocols, and discontinuation practices. Post-baseline gaps interventions included staff education and standardized checklists.

Results: Cycle 1 revealed critical deficiencies, which were as follows: nasal cannula at 2-6 L/min (26.0%; 8/30), non-rebreather mask at 10-15 L/min (17.4%; 5/30), weaning compliance (33.3%; 10/30), and target saturation documentation (46.7%; 14/30). Post intervention (Cycle 2), nasal cannula compliance reached 100% (30/30; +74%), non-rebreather mask 91.6% (27/30; +74.2%), and face mask 100% (30/30; +21.7%). Documentation of oxygen saturation improved to 100% (30/30; +10%), target saturation recording to 100% (30/30; +53.3%), and weaning compliance to 91.6% (27/30; +58.3%). Immediate oxygen for at-risk patients rose to 96.7% (29/30; +16.7%). Notably, timely discontinuation decreased to 76.7% (23/30; -13.3%), and the target range specification for type 2 failure remained suboptimal (66.7%; 20/30).

Conclusion: Education and protocol implementation significantly improved oxygen delivery accuracy and documentation. The decline in discontinuation compliance highlights an ongoing challenge requiring focused interventions. Sustained adherence necessitates regular reinforcement and re-auditing.

## Introduction

Acute oxygen therapy is a cornerstone intervention for managing critically ill and hypoxemic patients in emergency and inpatient settings [1]. International guidelines, especially those from the British Thoracic Society (BTS), highlight the importance of viewing and prescribing oxygen as a drug, with explicit indications, target saturation ranges, and comprehensive monitoring protocols to ensure patient safety [1-2]. The BTS 2017 guidelines recommend a target oxygen saturation range of 94% to 98% for most acutely ill adults and 88% to 92% for patients at risk of hypercapnic respiratory failure, with each prescription carefully documented and regularly monitored using pulse oximetry [2-4].

Despite the availability of such evidence-based recommendations, audits across healthcare institutions worldwide repeatedly reveal deficits in real-world practice: oxygen is frequently administered without a clear prescription, target saturations may not be specified, and documentation or monitoring of therapy can be inconsistent [3-5]. This lack of adherence underlines the critical need for regular clinical audits, which serve to benchmark current practices, identify areas for targeted improvement, and drive alignment with best-practice protocols [6-8]. Moreover, knowledge gaps and perceived barriers among healthcare professionals further complicate acute oxygen therapy delivery [9].

Atbara Teaching Hospital, located in Atbara, Sudan, implemented a structured clinical audit of acute oxygen therapy to provide valuable insights into current practice, illuminate areas where patient care can be further optimized, and guide the introduction of evidence-based interventions that enhance safety and treatment outcomes.

## Materials and methods

This quality improvement clinical audit was conducted at Atbara Teaching Hospital, a tertiary care center in Atbara, Sudan, with the aim of evaluating and improving the standards of acute oxygen therapy administered in the emergency department. A mixed retrospective and prospective observational design was used to capture real-world practice across two formal audit cycles. The study population comprised all patients who received oxygen therapy in the emergency department during the audit periods, with the first cycle conducted from 15^th^ September to 15^th^ October 2024, and the second cycle from 10^th^ June to 24^th^ June 2025.

Audit standards and criteria

The audit criteria and standards were based on the BTS Guideline for oxygen use in adults in healthcare and emergency settings (Appendix A) [1]. Audit parameters included the correct delivery of oxygen via nasal cannula at 2-6 L/min, face mask at 6-10 L/min, or non-rebreather mask at 10-15 L/min; the documentation of oxygen saturation on observation sheets; the recording of target oxygen saturation for patients with type 2 respiratory failure (88% to 92%); and documentation of target saturation in patient notes. Additional standards assessed proper adjustment or weaning of flow rates in stable patients, prompt disconnection after maintenance of adequate oxygen saturation, and immediate provision of oxygen for patients at risk of hypoxemia.

First cycle: baseline audit and problem identification

This audit was designed as a quality improvement project to assess and enhance oxygen therapy delivery through baseline evaluation, targeted education, protocol implementation, and re-audit. During the first audit cycle, patient charts and observation sheets were systematically reviewed for compliance with the established standards. Baseline data revealed significant gaps in care, which were as follows: only 26% of patients received a nasal cannula at the correct flow rate, and only 17.4% received a non-rebreather mask at the recommended rate. Documentation of target oxygen saturation for patients with type 2 respiratory failure was inconsistent (51.6%), and only 46.7% of cases had the target saturation written in patient notes. The audit also highlighted deficiencies in the proper weaning of oxygen (33.3% compliance) and timely disconnection after stabilization (90%). Informal staff interviews and documentation reviews identified insufficient guideline awareness and the absence of standardized checklists or protocols as major obstacles to optimal care.

Intervention: education and protocol implementation

Following root cause analysis, targeted interventions were implemented. Educational sessions were organized for emergency department staff, focused on promoting the core components of the BTS guideline: appropriate prescription practices, rationale for specific oxygen delivery modes and flow rates, the importance of documentation, and risks associated with inappropriate administration.

The intervention targeted frontline healthcare providers in the emergency department, specifically doctors and nurses directly responsible for initiating and monitoring oxygen therapy. On average, 20 to 22 staff members participated regularly in the program.

In May 2025, two structured educational sessions (two hours each) were conducted in the emergency department conference room. These sessions focused on oxygen delivery modalities, safe titration and weaning protocols, and proper documentation practices. In addition, two practical workshops (three hours each) were delivered, emphasizing hands-on skill training, case-based discussions, and troubleshooting of common oxygen delivery challenges.

Second cycle: post-intervention audit and outcome assessment

The second audit cycle utilized the same population, criteria, and assessment methods, with data collected from 10^th^ to 24^th^ June 2025. Marked improvements were observed across most parameters, which were as follows: use of nasal cannula and face mask at proper flow rates reached 100%, while non-rebreather mask delivery improved to 91.6%. Documentation of oxygen saturation rose to 100% compliance, and proper weaning in stable patients increased to 91.6%. Provision of immediate oxygen for at-risk patients improved to 96.7%. Notably, there was a minor decrease in the documentation of target saturations in patient notes by treating doctors (down to 26.7%), indicating an area for continued focus. These changes affirmed the utility of audit, education, and real-time protocol reminders in driving compliance with best practice and improving the quality of oxygen therapy.

Data collection

Patient case sheets were reviewed prospectively using a standardized audit proforma adapted from the World Health Organization and BTS guidelines. Each case sheet was assessed for completeness of documentation and accuracy of oxygen delivery. Ethical approval was obtained from the local Institutional Review Board of Atbara Teaching Hospital (approval number: 24/AU/0565), and administrative permission for data collection was formally granted by the hospital administration. Data extraction was carried out by the research team under supervision, ensuring uniformity of assessment.

Evaluation of impact

Staff performance was evaluated through practice-based assessments embedded in the audit cycles. The first cycle (September to October 2024) provided the baseline evaluation of skills and practice, while the second cycle (June 2025) served as the post-intervention evaluation. Comparison of these cycles allowed measurement of the impact of the educational and protocol-driven interventions.

## Results

A total of 30 patient records were reviewed during both the first and second audit cycles. Overall, there was a clear improvement in adherence to oxygen delivery and documentation guidelines following the educational intervention. Statistical comparison using chi-square tests between the two cycles demonstrated significant improvements across most parameters. Table 1 summarizes these significant improvements in adherence to oxygen therapy protocols between the two audit cycles.

**Table 1:** Improvement in oxygen therapy adherence between first and second audit cycles Data are represented as N (number) and % (percentage). "Gap to 100%":  percentage difference from perfect compliance in the first cycle; χ²: chi-square statistic; statistical significance was set at p<0.05.

Parameter	First cycle N (%)	Second cycle N (%)	Gap to 100% (Cycle 1)	χ² Value	p-value
Received oxygen via nasal cannula (2–6L/min)	8 (26.7%)	30 (100.0%)	73.30%	34.74	<0.001
Received oxygen via non-rebreather mask (10–15L)	5 (16.7%)	27 (90.0%)	83.30%	32.41	<0.001
Received oxygen via face mask (6–10L/min)	23 (76.7%)	30 (100.0%)	23.30%	7.92	0.005
Oxygen saturation (SpO₂) recorded on the observation sheet	27 (90.0%)	30 (100.0%)	10.00%	3.16	0.076
Target SpO₂ type 2 respiratory failure (88%–92%)	15 (50.0%)	20 (66.7%)	50.00%	1.71	0.19
Target SpO₂ written in patient notes	14 (46.7%)	30 (100.0%)	53.30%	21.82	<0.001
Oxygen flow rate reduced (weaning protocol)	10 (33.3%)	27 (90.0%)	66.70%	20.38	<0.001
Disconnected once stable	27 (90.0%)	23 (76.7%)	10.00%	1.92	0.166
Immediate O₂ for high-risk hypoxemia	24 (80.0%)	29 (96.7%)	20.00%	4.04	0.044

The most pronounced improvement was observed in the use of nasal cannula for oxygen delivery at 2-6 L/min, which increased from eight patients (26.0%) in the first cycle to 30 patients (100%) in the second cycle, representing a 74.0% absolute increase. Similarly, the use of a non-rebreather mask at the correct flow rate rose from five patients (17.4%) to 27 patients (91.6%), an improvement of 74.2%. The use of face masks at 6-10 L/min improved from 23 patients (78.3%) to full compliance at 30 patients (100%), a gain of 21.7%. Documentation of oxygen saturation on observation sheets also reached 100% in the second cycle, up from 27 patients (90.0%), effectively closing the documentation gap.

Significant gains were recorded in documenting the target oxygen saturation for patients with type 2 respiratory failure, which increased from complete documentation in 14 patients (46.7%) to 30 patients (100%), a 53.3% improvement. Compliance with the recommendation to reduce oxygen flow rate in stable patients (weaning) also substantially improved, rising from 10 patients (33.3%) to 27 patients (91.6%), a 58.3% increase.

Moderate improvement was observed in the correct identification of the target oxygen saturation range (88% to 92%) for patients with type 2 respiratory failure, rising from 15 patients (51.6%) to 20 patients (66.7%), a 15.1% increase. The ability to rapidly deliver oxygen to patients at risk of hypoxemia improved from 24 patients (80.0%) to 29 patients (96.7%), a 16.7% increase.

However, a few parameters showed minimal improvement or an unexpected decline. The proportion of patients disconnected from oxygen once able to maintain saturation fell from 27 patients (90.0%) in the first cycle to 23 patients (76.7%) in the second, a decrease of 13.3%, highlighting the need for continued focus on weaning protocols.

In summary, educational interventions led to marked improvements in most aspects of oxygen therapy practice among staff, particularly regarding documentation and correct delivery modalities. Nonetheless, ongoing gaps remain in tailoring and discontinuing oxygen therapy, indicating the need for further quality improvement initiatives. Regular education, protocol reinforcement, and repeat audits are essential to sustain and enhance adherence to best practice guidelines.

## Discussion

Acute oxygen therapy is a cornerstone intervention in the management of critically ill and hypoxemic patients, both in emergency and inpatient settings. International guidelines, particularly from the BTS, underscore the principle that oxygen should be prescribed and monitored akin to any other medication, with clear indications, defined target saturation ranges, and consistent documentation to ensure patient safety [1-2]. The BTS 2017 guidelines specifically advocate for a target oxygen saturation range of 94% to 98% for the majority of acutely ill adults and 88% to 92% for those at risk of hypercapnic respiratory failure, recommendations that are only effective when clearly documented and routinely monitored with pulse oximetry [2-4].

Our audit at Atbara Teaching Hospital revealed substantial gaps in the quality of oxygen therapy administration and documentation during the first cycle. For example, only 26.0% of patients received oxygen via nasal cannula at the recommended rate, and just 46.7% had target saturations written in the medical notes by the treating doctor. There was also significant underdocumentation for patients with type 2 respiratory failure; only about half (51.6%) had appropriate target saturations documented, and weaning practices lagged far behind best-practice standards (Table 1).

Following the educational and protocol-based interventions, statistical comparison using chi-square tests between the first and second audit cycles demonstrated significant improvements across multiple parameters, confirming the impact of the intervention beyond simple percentage changes. For example, correct nasal cannula use increased from 26.7% to 100% (χ² = 34.74, p < 0.001), and compliance with weaning protocols rose from 33.3% to 91.6% (χ² = 20.38, p < 0.001). Documentation of target oxygen saturation for type 2 respiratory failure improved from 46.7% to 100% (χ² = 21.82, p < 0.001) (Table 1). These findings are consistent with international evidence that structured audit-feedback cycles and staff training are effective strategies to improve adherence to oxygen therapy guidelines [3-5].

Despite these improvements, certain deficiencies persisted. Comprehensive documentation of target saturations for patients with type 2 respiratory failure rose but remained suboptimal at 66.7%. In addition, a small decline was seen in the percentage of patients being disconnected from oxygen after achieving stable saturations (from 90% to 76.7%), indicating the need for sustained focus on safe de-escalation and discharge practices. Such gaps are not unique to our institution; they reflect widely reported challenges in translating guidelines into routine care, as shown in similar audits across healthcare systems globally [4-5]. Additionally, knowledge gaps and perceived barriers among healthcare professionals have been identified as key factors complicating adherence to optimal oxygen therapy practices [9].

While these improvements were expected, the magnitude and consistency across multiple parameters highlight the effectiveness of structured educational sessions and protocol implementation in enhancing staff skill and practice. We acknowledge the possibility of bias, including the Hawthorne effect, where staff behavior may temporarily improve due to awareness of being observed during data collection. Staff turnover was minimal, and the core group receiving training remained largely constant, mitigating this potential bias.

The critical role of continuous clinical audit is highlighted by these results; not only does it identify deviations from evidence-based practice, but it also creates accountability and a platform for ongoing education [6-8]. Multidisciplinary engagement, easy access to summarized guidelines at the point of care, and process reminders within clinical documentation (such as discharge cards) are all recommended to help reinforce and maintain high standards [6-8].

Limitations of this audit include its single-center setting and a relatively small sample, which may affect generalizability. Although staff performance was evaluated through skill and practice in the audit cycles, formal pre- and post-training skills checklists were not used, which could have provided additional rigor. Additionally, process measures were emphasized over patient-centered clinical outcomes, such as morbidity or length of stay. Future audits should ideally incorporate such data to more fully evaluate the clinical impact of improved oxygen therapy practices. A further limitation was that staff perceptions were explored through informal interviews rather than a structured knowledge, attitude, and practice (KAP) questionnaire. Incorporating a validated KAP tool in future audits would provide a more systematic and quantifiable assessment of staff knowledge and barriers, thereby strengthening the design of targeted interventions.

## Conclusions

This audit highlights that structured, protocol-driven interventions combined with regular education and real-time feedback can lead to substantial improvements in the safe prescription, delivery, and documentation of acute oxygen therapy. Our findings demonstrate that adherence to internationally recognized guidelines, such as the BTS recommendations, significantly enhances clinical practice in oxygen administration. However, despite notable progress after implementing corrective measures, some deficiencies persist, particularly in documentation for patients with type 2 respiratory failure and timely discontinuation of oxygen therapy, which require ongoing attention. These gaps emphasize the necessity of sustained efforts, including continuous clinical audits, multidisciplinary collaboration, and the integration of easily accessible guidelines into routine workflows.

Regular reinforcement of staff training and quality improvement cycles remains essential to maintaining and further advancing compliance with best-practice standards. Given the critical role oxygen therapy plays in patient safety and outcomes, especially in low-resource settings like ours, robust monitoring and evaluation mechanisms should be institutionalized. Future audits incorporating patient-centered clinical outcomes such as morbidity and length of hospital stay will provide a more comprehensive evaluation of intervention impact. Overall, fostering a culture of continuous improvement and evidence-based practice is vital to ensuring optimal and equitable oxygen therapy delivery, ultimately improving patient care and safety.

## Appendices

Appendix A 

**Table 2:** Oxygen therapy compliance audit questionnaire Source: [1]

No.	Question	Response (Yes/No)	Comments
1	Did the patient receive oxygen via nasal cannula at a rate of 2–6 L/min?	☐ Yes ☐ No	
2	Did the patient receive oxygen via a non-rebreather mask at a rate of 10–15 L/min?	☐ Yes ☐ No	
3	Did the patient receive oxygen via a face mask at a rate of 6–10 L/min?	☐ Yes ☐ No	
4	Was the oxygen saturation recorded on the observation sheet?	☐ Yes ☐ No	
5	For patients with type 2 respiratory failure, was the target SpO₂ maintained between 88–92%?	☐ Yes ☐ No	
6	Was the target oxygen saturation documented in the patient's notes by the treating doctor?	☐ Yes ☐ No	
7	Was the oxygen flow rate reduced (weaning) for stable patients?	☐ Yes ☐ No	
8	Was oxygen discontinued once the patient could maintain their oxygen saturation without support?	☐ Yes ☐ No	
9	Was oxygen delivered immediately to patients at risk of hypoxemia?	☐ Yes ☐ No	
